# Genome wide transcriptome analysis provides bases on hepatic lipid metabolism disorder affected by increased dietary grain ratio in fattening lambs

**DOI:** 10.1186/s12864-023-09465-4

**Published:** 2023-06-29

**Authors:** Hui Mi, Fan Hu, Kefyalew Gebeyew, Yan Cheng, Ruiping Du, Min Gao, Zhixiong He, Zhiliang Tan

**Affiliations:** 1 CAS Key Laboratory for Agro-Ecological Processes in Subtropical Region, National Engineering Laboratory for Pollution Control and Waste Utilization in Livestock and Poultry Production, Hunan Provincial Engineering Research Center for Healthy Livestock and Poultry Production, Institute of Subtropical Agriculture, South-Central Experimental Station of Animal Nutrition and Feed Science in Ministry of Agriculture, The Chinese Academy of Sciences, Changsha, 410125 China; 2 University of Chinese Academy of Sciences, Beijing, 100049 China; 3 Inner Mongolia Academy of Agricultural and Animal Husbandry Sciences, Hohhot, Inner Mongolia 010031 China

**Keywords:** Hepatic lipid metabolism, Fattening lamb, Dietary grain ratio, RNA-sequencing

## Abstract

**Background:**

The liver is a principal metabolic organ and has a major role in regulating lipid metabolism. With the development of rapidly fattening livestock in the modern breeding industry, the incidence of hepatic steatosis and accumulation in animals was significantly increased. However, the molecular mechanisms responsible for hepatic lipid metabolic disturbances in a high concentrate diet remain unclear. The objective of this study was to evaluate the effects of increasing concentrate level in a fattening lamb diet on biochemical indices, hepatic triglycerides (TG) concentration, and hepatic transcriptomic profiles. In the present study, 42 weaned lambs (about 3 ± 0.3 months old) were randomly assigned to the GN60 group (60% concentrate of dry matter, GN60, n = 21) or GN70 group (70% concentrate of dry matter, n = 21) for a 3-months feeding trial.

**Results:**

No difference was observed in the growth performance or plasma biochemical parameters between the GN60 group and the GN70 group. The hepatic TG concentration was higher in the GN70 group than GN60 group (P < 0.05). Hepatic transcriptomic analysis showed that there were 290 differentially expressed genes identified between GN60 and GN70 groups, with 125 genes up-regulated and 165 genes down-regulated in the GN70 group. The enriched Gene Ontology (GO) items and KEGG pathways and protein–protein interaction (PPI) network of differentially expressed genes (DEGs) revealed that the majority of enriched pathways were related to lipid metabolism. Further analysis revealed that the fatty acid synthesis was up-regulated, while fatty acid transport, oxidation, and TG degradation were down-regulated in the GN70 group when compared with the GN60 group.

**Conclusions:**

These results indicated that GN70 induced excess lipid deposition in the liver of lambs during the fattening period, with high synthesis rates and low degradation rates of TG. The identified mechanisms may help understand hepatic metabolism in lambs with a high concentrate diet and provide insight into decreasing the risk of liver metabolism disorder in animals.

**Supplementary Information:**

The online version contains supplementary material available at 10.1186/s12864-023-09465-4.

## Introduction

Mutton is high-protein, low-fat and low-cholesterol features and has become a primary source of dietary meat. In the current intensive production systems, increasing the proportion of concentrate-roughage in the ruminant diet to provide sufficient energy for growing and reaching the desired slaughter weight in a short period has become a popular feeding pattern [1]. However, it worth noting that despite improving the ratio of concentrate to forage (F: C) can increase the levels of weight gain and/or efficiency of gain, there may be a change in the metabolic state of animals [2]. Previous studies have reported that dietary nutrient levels such as F: C significantly affected animal metabolic activities [3, 4]. Research has consistently shown that feeding a high concentrate diet in ruminants can lead to rapid fermentation and subsequent excessive organic acid accumulation. Such animals are likely to suffer such as subacute ruminal acidosis, and disruptions of the mucosal barrier [5–7]. Moreover, several researchers found that an excessive carbohydrate concentrate ratio diet induced lipid metabolism disorders and promoted ectopic lipid accumulation [8, 9].

The liver is a pivotal metabolic organ of lipid, which can directly and clearly reflect the abnormal lipid metabolism [10]. Ectopic lipid metabolism and accumulation can lead to a spectrum of liver diseases which range from steatosis to non-alcoholic steatohepatitis, cirrhosis, and hepatocellular carcinoma, resulting in reduced animal production capacity [11]. One caveat is that, for a long time, researchers have used the changes in blood and liver biochemical indicators [such as triglycerides (TG) and Cholesterol (TC)] to characterize disorders in hepatic lipid metabolism and have focused on specific genes to interpret it based on their expression [2, 12, 13]. These designs do not provide a comprehensive range for predicting lipid metabolism status, and the molecular mechanisms underlying lipid metabolism regulation remains unclear.

Hepatic lipid metabolism involves a complex network of interrelated metabolic pathways, including lipid synthesis, lipolysis, oxidation, and secretion [14]. Changes in gene expression and enzyme activity associated with these processes may result in changes in the lipid flux of the liver [15, 16]. Therefore, to explore the transformation of hepatic gene expression profile with an excessive carbohydrate concentration ratio diet may improve our understanding of its regulating mechanism at a molecular level. With the development of sequencing technologies, RNA-sequencing (RNA-seq) has emerged as a practical approach that enables the interrogation of the whole transcriptome [17]. This method provides sensitive and unbiased detection of all expressed genes and has been successfully applied to hepatic transcripts associated with lipid metabolism in sheep [17, 18], cattle [19, 20], poultry [21] and pigs [22]. The objective of this study was to evaluate the effects of increasing concentrate level (from 60 to 70%) in a fattening lamb diet on lipid metabolism related biochemical indices, and hepatic transcriptomic profiles.

## Materials and methods

### Animal management and dietary treatments

Forty-two healthy Weaned Hulun Buir lambs with an average initial body weight (IBW) of 16.84 ± 0.37 kg (about 3 ± 0.3 months old), were used in this trial. All lambs were blocked by IBW and then randomized into 6 pens (7 lambs per pen). Pens were randomly assigned to one of the following two diets: (1) a diet containing 60% corn and barley based concentrate and 40% alfalfa hay (GN60), (2) a diet containing 70% corn and barley based concentrate and 30% alfalfa hay (GN70). At present, based on the unique digestive characteristics of ruminants, sheep farms currently use a typical 60% concentrate diet as lamb fodder [23]. Therefore, a concentrate ratio of 60% was used in the control group (i.e., GN60). The ingredients and nutrient composition of the experimental diets were determined as described in Additional file 1. The animal house was well ventilated, and before the experiment, the floor, walls, and fences of the sheep house were thoroughly disinfected. During the preliminary experiment period, the health of lambs was evaluated, and all lambs were treated for parasites.

Lambs in each group undergone a 14 day adaptation period, and then the formal experimental period was 3 months. Animals were fed the respective diets three times daily at 06:00 am, 11:00 am and 18:00 pm, and had free access to water during the experimental period. The amount of feed intake was recorded daily to determine the average daily feed intake (ADFI).

### Sample collection

At the end of the feeding experiment, blood samples were collected before morning feeding using EDTA-containing vacuum tubes from the jugular vein and centrifuged for 10 min at 3000 ×g to separate plasm, then stored at − 80 °C for subsequent analysis. Seven sheep in each group were then randomly selected for slaughter and fasted for 12 h before slaughter. Pre-slaughter live weight was determined and recorded to investigate the average daily gain (ADG). Within 20 min after slaughter, samples were collected from the same site in the liver and immediately stored in liquid nitrogen and then stored at − 80 °C until RNA sequencing, quantitative real-time PCR, and biochemical indicators detection.

### Plasma biochemical parameters

The concentrations of glucose (GLU), TC, TG, low-density lipoprotein cholesterol (LDL-C), and high-density lipoprotein cholesterol (HDL-C) in the blood and hepatic were measured by automatic chemistry analyzer Roche Cobas c311 (Roche, Switzerland), following the machine’s instructions. Non-esterified fatty acid (NEFA) and β-hydroxybutyric acid (BHBA) levels in blood were measured with the ELISA kits (MEIMIAN, Jiangsu, China).

### Liver glucose, triglycerides, and cholesterol activity

Approximately 200 mg liver tissue was homogenized in ice-cold phosphate buffer saline (pH 7.4, Solarbio LIFE SCIENCES, Beijing, China) using a freezing grinder (KZ-5 F-3D, Servicebio, Wuhan, China) was used to suspend the homogenate. Subsequently, the homogenate was centrifuged at 12 000 rpm for 15 min at 4 ℃, collected supernatant. The GLU, TG, and TC levels were determined using an automatic chemistry analyzer Roche Cobas c311 (Roche, Switzerland).

### RNA sequencing of hepatic tissues

Total RNA was extracted from the hepatic tissues using Trizol (Invitrogen, Carlsbad, CA, USA) according to the manufacturer’s protocols. Then total RNA concentration and purity were quantified by the NanoDrop and Agilent 2100 bioanalyzer (Thermo Fisher Scientific, MA, USA). Meanwhile, RNA integrity was observed by agarose gel electrophoresis. When the absorption ratio of A260/A280 was between 1.9 and 2.0, the proportion of 28 S/18S ranged from 1.8 to 2.0, and the integrity number was between 7 and 10, an RNA sample was deemed to be sufficiently high quality. In the present study, all of the hepatic samples have shown high-quality RNA available for transcriptome sequencing.

Hepatic mRNA was further purified with Oligo(dT) magnetic beads and then fragmented in fragment buffer at the appropriate temperature. Subsequently, the first and second strands of cDNA were synthesized, purified, and end repaired. PCR amplified the cDNA fragments, purified products by Ampure XP Beads, then dissolved in EB solution. The products from the previous step were heated, denatured, and circularized by the splint oligo sequence to get the final library. The final library was amplified with phi29 to make a DNA nanoball (DNB). DNBs were loaded into the patterned nanoarray, and single-end 50 bases read generated on the BGIseq 500 platform (BGI-Shenzhen, China).

The sequencing data was filtered with SOAPnuke (v1.5.2) [24]. Specifically, reads containing sequencing adapters read with low-quality base ratio (base mass less than or equal to 5) greater than 20% and reads with unknown base (‘N’ base) ratio greater than 5% are removed to obtain subsequent clean reads. Clean reads were mapped to the Ovis Aries reference genome (Oar V3.1) and aligned to the reference coding gene set. Finally, transcription fragment per million fragments per thousand bases (FPKM) was calculated based on the number of matched reads and transcript length to assess gene expression levels.

### Identification of differentially expressed genes and functional enrichment analysis

The identification of differentially expressed genes (DEGs) between GN60 and GN70 was performed by DESeq2 (V1.4.5) [25]. Thresholds with a false discovery rate (FDR) < 0.05 (Q Value < 0.05)and an absolute value of |fold change| ≥ 1.5 were set to filter differentially expressed genes (DEGs). To take an insight into the shift of Gene Ontology (GO) items and KEGG pathways [26], enrichment analysis of DEGs was performed by Phyper based on Hypergeometric test (https://en.wikipedia.org/wiki/Hypergeometric_distribution). The significant levels of pathways were corrected by FDR < 0.05 (Q Value < 0.05) by Bonferroni [27]. Given that the search of protein interactions and the interaction network is the critical procedure for gaining the insights into cellular organization, bioprocess, and functions, DEGs were submitted to the online search tool STRING database2 to search for interacting genes to obtain gene interaction relationship and the protein–protein interaction (PPI) network. The interaction confidence score was set to 0.4, other parameters were set to default.

### Validation of RNA-sequencing data

Total RNA was extracted from the liver tissue of each sheep was using a commercial kit (AG21017, Accurate Biology, Changsha, China), following the method provided by manufacturer, and the RNA was dissolved in DNase/RNase free dH_2_O. The concentration and quality of total RNA were evaluated through a NanoDrop 2000 spectrophotometer (Thermo Scientific, Waltham, MA, USA). The extracted RNA with a 260/280 nm ratio in the range 1.8 ~ 2.0 were considered high quality and selected for further analysis. The extracted RNA was reverse transcribed using a cDNA reverse transcription kit (AG11705, Accurate Biology, Changsha, China). Subsequently, real-time quantitative PCR (RT-qPCR) was performed with a LightCycler 480 II Instrument (Roche, Basel, Switzerland) using an SYBR® Green Premix Pro Taq HS qPCR Kit (Accurate Biology, Changsha, China).

The reaction volume for PCR was 10 µL, including 5 µL SYBR, 0.2 µL of 10 µM forward or reverse primer, 1 µL cDNA, and 3.6 µL dH2O. The qPCR conditions consisted of a hold stage at 95 °C for 30 s, followed by 40 cycles of 95 °C for 5 s, 60 °C for 30 s, and a melt curve of increasing temperature of 0.5 °C every 5 s starting at 65 − 95 °C. The primers of target genes were designed by Premier Primer 5 software (PREMIER Biosoft International, CA, USA), and the details of the primer sets are provided in Additional file 2. The expression values of all target genes in the hepatic were determined and normalized against housekeeping genes (GAPDH and β-actin) and internal GN60 group using a 2^–ΔΔCt^ method [28].

### Statistical analysis

The data for the relative gene expression and biochemical indicators between two groups were all performed using the independent-samples *t*-test in SPSS 22.0. All data were presented as mean ± SEM, and significance was declared at *P* < 0.05.

## Result

### Growth performance and biochemical indicators

The growth performance of lamb fed different diets is presented in Table 1. The ADG, ADFI, and feed to gain ratio (F/G) in GN70 were similar with those observed for the GN60 group. The concentrations of GLU, TG, TC, LDL, HDL, NEFA and BHBA in blood were similar between the GN60 and GN70 groups (Table 2). There was no difference between two groups observed for hepatic GLU and TC, expect that GN70 had a higher TG concentration (*P* = 0.01, Table 3).

**Table 1:** Effect of increasing dietary grain ratio on growth performance of fatten lambs

^1^Item	Group		*P*-vaule
	GN60	GN70	
Initial BW, kg	17.12 ± 0.388	17.92 ± 0.631	0.318
ADG, g/d	176.40 ± 2.469	178.07 ± 4.427	0.438
ADFI, kg/d	1.31 ± 0.045	1.25 ± 0.059	0.763
F/G, g/g	7.45 ± 0.167	7.05 ± 0.114	0.132

^1^BW = body weight; ADG = average daily gain; ADFI = average daily feed intake; F/G = feed/gain ratio

Values are means ± SEM.

**Table 2:** Effect of increasing dietary grain ratio on biochemical parameters in plasma of lambs

^1^Item	Group			*P*-Value
	GN60		GN70	
GLU(mmol/l)		4.54 ± 0.211	4.11 ± 0.193	0.160
TG(mmol/l)		0.30 ± 0.029	0.31 ± 0.011	0.741
TC(mmol/l)		2.08 ± 0.216	2.04 ± 0.164	0.906
LDL(mmol/l)		0.75 ± 0.126	0.72 ± 0.098	0.837
HDL(mmol/l)		1.03 ± 0.085	1.17 ± 0.093	0.327
NEFA(mmol/l)		1.10 ± 0.097	0.92 ± 0.090	0.187
BHBA(mmol/l)		0.53 ± 0.089	0.52 ± 0.056	0.927

^1^GLU= Glucose; TG = triglyceride; TC = total cholesterol; LDL = low-density lipoprotein holesterol; HDL = high-density lipoprotein holesterol; NEFA = non-esterified fatty acids; BHBA = β-hydroxybutyric acid

Values are means ± SEM.

**Table 3:** Effect of increasing dietary grain ratio on hepatic biochemical parameters lambs

^1^Item		Group			*P*-Value
		GN60		GN70	
GLU (µmol/g)	76.08 ± 10.982			88.17 ± 5. 346	0.303
TG (µmol/g)			7.64 ± 0.762	11.41 ± 0.864	0.011
TC (µmol/g)			2.86 ± 0.323	2.81 ± 0.361	0.923

^1^GLU= Glucose; TG = triglyceride; TC = total cholesterol

Values are means ± SEM.

### Characterization of the liver transcriptome in liver

**Fig. 1:**
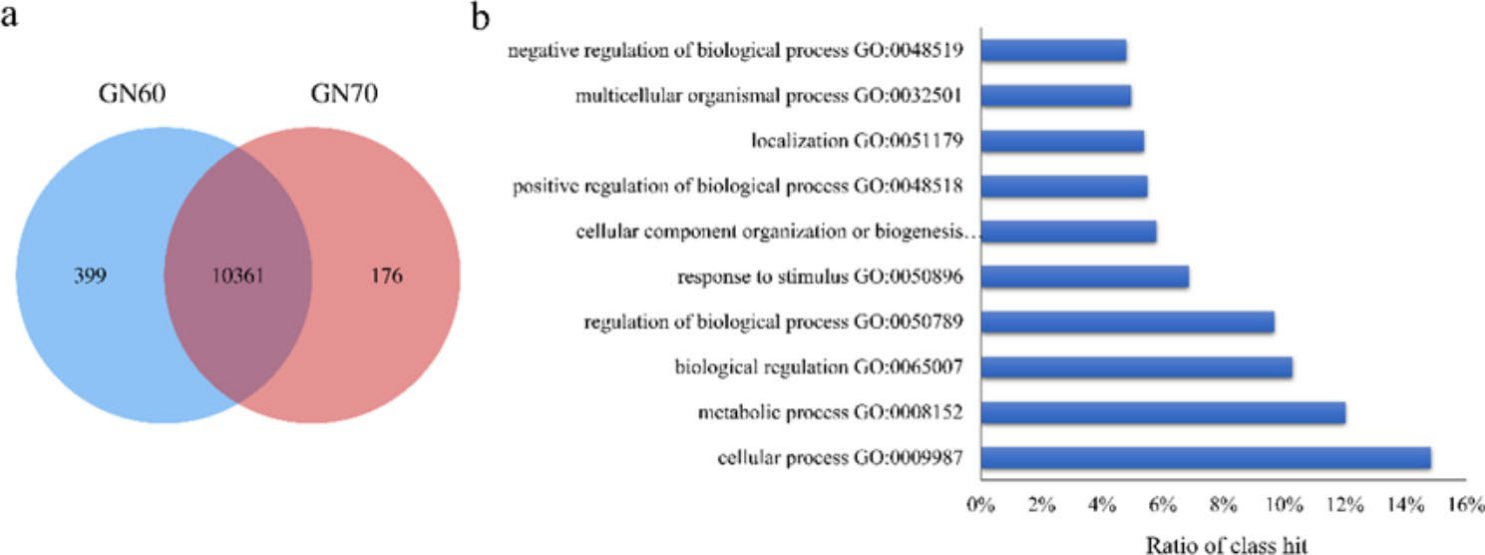
Hepatic core transcriptomic profiles in fattening lambs. **a** Venn diagram of expressed genes in livers of GN60 and GN70. **b** Biological processes of core genes

In total, 334,922,896 reads were generated by the 14 liver samples, with 23,923,064 reads per sample. The number of expressed genes was 16,373 ± 37 in liver regardless of treatments (Additional file 3), and 10,361 of genes were expressed (FPKM) > 1 in all animals which were defined as core transcriptome (Fig. 1a, Additional file 4). The functional analysis of the core transcriptome revealed 28 biological processes in total, with “cellular process” (14.85%), “metabolic process” (12.03%) and “biological regulation” (10.28%) being the three predominant biological processes (Fig. 1b). When the transcriptome profiles were further compared between diets, the diet-dependent expression of genes was detected, with 399 and 176 genes being specifically expressed in GN60 and GN70 grain, respectively (Fig. 1a, Additional file 5). Further analysis of the diet-dependent genes using DAVID (Database for Annotation, Visualization, and Integrated Discovery) showed different functional annotation clusters (Additional file 5). The enriched gene ontologies for 399 genes in GN60 and 176 genes in GN70 were “DNA replication initiation” and “immunological synapse formation”, respectively.

### Differential expressed genes in liver

**Fig. 2:**
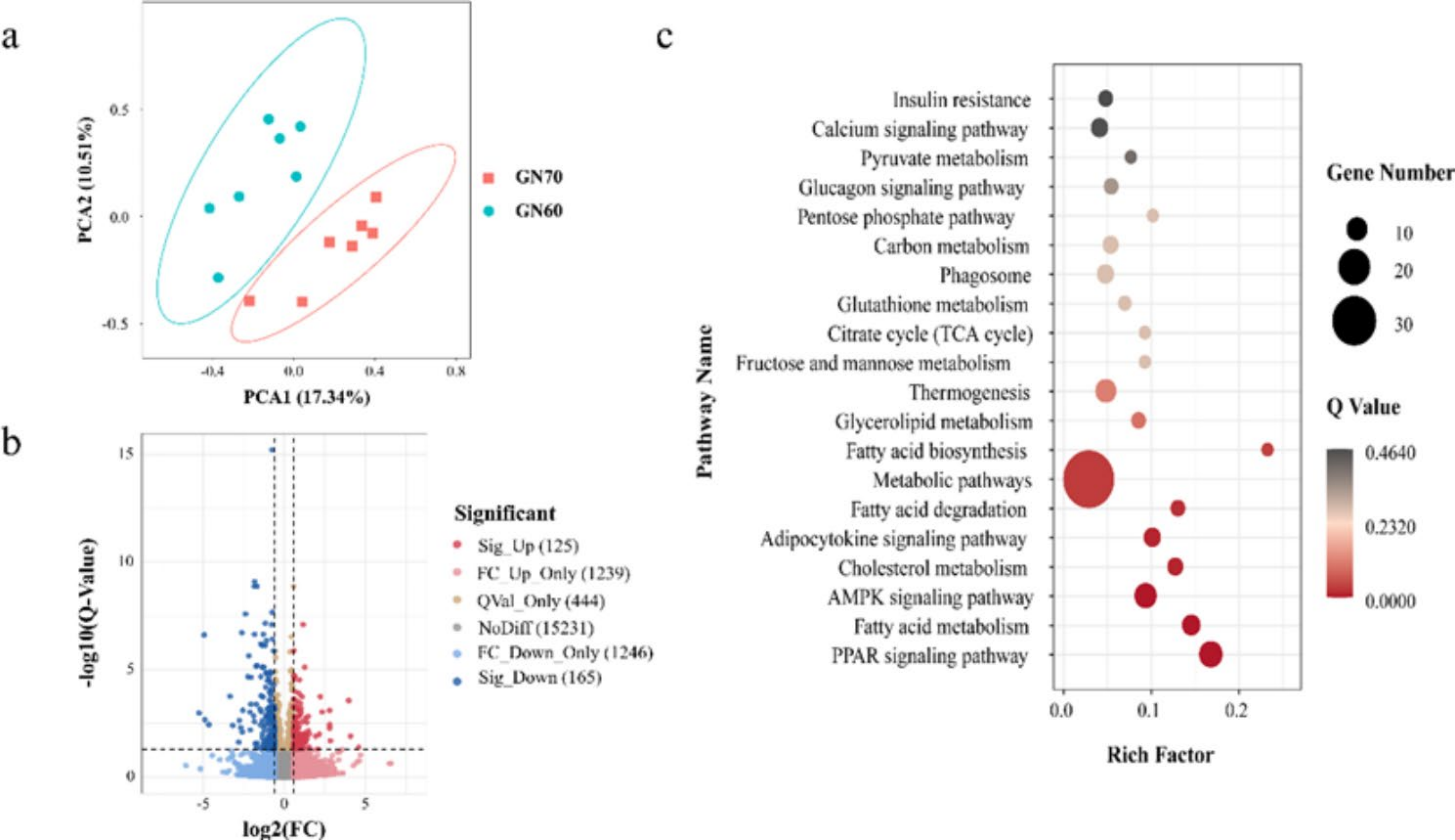
Transcriptomic differences between GN60 and GN70. **a** Principal component analysis of the total detected genes fed GN60 and GN70 diets. The X and Y-axis represent the first two principal components. The percentage value in the bracket represents the percentage of variance explained by that principal component. **b** Volcano Plot of up- and down- regulated DEGs between GN60 and GN70 group. Sig_Up: significantly up-regulated; FC_Up_Only: The difference is up-regulated, but the p value is not significantly different; QVal_Only: significant difference in Q value, but insignificant difference multiple; NoDiff: No difference; FC_Down_Only: The difference is down, but the Q value is not significantly different; Sig_Down: significant difference down. **c** KEGG pathway enrichment highlights the markedly altered lipid metabolism correlation pathway. Circle size indicates the number of DEGs enriched in the pathway, and circle colour indicates the degree of enrichment

Total genes’ principal component analysis (PCA) displayed clear separation between the GN60 and GN70 groups (Fig. 2a). Based on the criteria of Q value < 0.05 and |fold change| ≥ 1.5, there were 290 DEGs identified between GN60 and GN70 groups, with 125 genes up-regulated and 165 genes down-regulated in GN70 group (Fig. 2b). Subsequently, KEGG and GO enrichment analysis were performed on DEGs to further confirm the regulation of biological processes. As shown in Fig. 2c, the majority of enriched KEGG pathways were related to lipid metabolism, including PPAR signaling pathway, fatty acid metabolism, fatty acid degradation, and fatty acid biosynthesis. Likewise, GO items of DEGs mainly included positive regulation of triglyceride catabolic process, positive regulation of lipoprotein lipase activity, and positive regulation of lipid catabolic process (Fig.S1). In addition, the genes related to the TOP3 GO items, including ABHD5, APOA5 and APOA4, were down-regulated. Protein–protein interaction network analysis was also performed on DEGs, we could get more insight into the interaction relationship among them. The interaction relationship of DEGs was shown in Figure S2A. We built the top three critical modules in DEGs’ PPI network through MCL clustering (Figure S2B, C, D). The top three modules’ genes were significantly enriched in DNA replication (FDR = 0.021), Fatty acid degradation (FDR = 0.008), and Cholesterol metabolism (FDR = 0.014).

### Differential expressed genes related to lipid metabolism in liver

The hepatic transcriptome analysis indicated that DEGs were enriched the lipid metabolism-related pathway. In order to further investigate the effect of a high concentrate diet inclusion on hepatic lipid metabolism, we screened the genes correlating with lipid metabolism from the liver transcriptome. There were 20 DEGs which were mainly classified in lipid metabolism and transport identified (Table 4). Genes involved in glycerolipid metabolism, such as Glycerol Kinase (GK), Monoglyceride Lipase (MGLL) and Lipase G (LIPG), were down-regulated, while Triokinase and Fmn Cyclase (TKFC) was up-regulated. Regarding fatty acid biosynthesis, the fatty acid synthase (FASN) was up-regulated. The genes for fatty acid oxidation including carnitine palmitoyltransferase (CPT) 1 alpha (CPT1A), CPT1B and CPT2 were down-regulated. For the genes in fatty acid transport, Acyl-Coa Synthetase Long Chain Family Member (ACSL) 1 was down-regulated, while ACSL3 showed increased expression. For the gene involved in lipid transport, Apolipoprotein A (APOA) 2 was up-regulated whereas APOA5 was down-regulated. Moreover, the up-regulation occurred for Ebp Cholestenol Delta-Isomerase (EBP) which was involved in Steroid biosynthesis.

**Table 4:** DEGs related to lipid metabolism in hepatic tissues

Gene Symbol	Gene Name	QValue	log2FC	Regulated
Glycerolipid metabolism				
GK	Glycerol Kinase	0.002	-1.350	Down
MGLL	Monoglyceride Lipase	< 0.001	-1.092	Down
LIPG	Lipase G, Endothelial Type	0.004	-3.174	Down
TKFC	Triokinase and Fmn Cyclase	0.002	0.683	Up
Fatty acid biosynthesis				
FASN	Fatty Acid Synthase	0.003	1.391	Up
Fatty acid elongation				
ELOVL5	Elovl Fatty Acid Elongase 5	0.027	-0.742	Down
TECR	Trans-2,3-enoyl-CoA reductase	< 0.001	0.59	Up
Fatty acid oxidation				
CPT1A	Carnitine Palmitoyltransferase 1 A	< 0.001	-0.923	Down
CPT1B	Carnitine Palmitoyltransferase 1B	0.034	0.662	Down
CPT2	Carnitine Palmitoyltransferase 2	0.004	0.638	Down
Fatty acid transport				
ACSL1	Acyl-Coa Synthetase Long Chain Family Member 1	0.049	-0.622	Down
ACSL3	Acyl-Coa Synthetase Long Chain Family Member 3	< 0.001	0.597	Up
Lipid transport				
APOA2	Apolipoprotein A2	0.026	0.594	Up
APOA5	Apolipoprotein A5	0.001	-0.981	Down
Steroid biosynthesis				
EBP	Ebp Cholestenol Delta-Isomerase	0.007	0.692	Up
Others				
CYP2J	Cytochrome P450, Family 2, Subfamily J	0.034	0.662	Up
CYP1A1	Cytochrome P4501A1	0.007	-0.642	Down
GPD1L	glycerol-3-phosphate dehydrogenase 1 like	< 0.001	-0.764	Down
GPX7	Glutathione peroxidase 7	0.020	-0.595	Down
SPHK2	Sphingosine Kinase 2	0.008	0.604	Up

FC, the mean value ratio of FPKM (fragments per kilobase of transcript per million fragments mapped, GN70/GN60).

### RT-qPCR validation

**Fig. 3:**
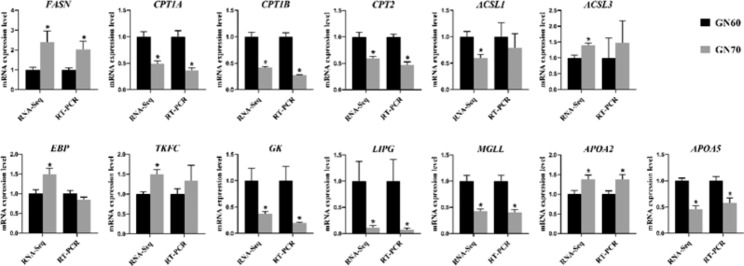
Validation of RNA-sequencing data with RT-qPCR. In RNA-sequencing data, mean values of FPKM with asterisks indicate a significant difference (Q < 0.05) between the two groups. In real-time quantitative PCR data, mean values of relative expression quantity with asterisks indicate significant differences (*P* < 0.05) RNA-Seq, RNA sequencing; RT-PCR, real-time quantitative PCR.

The expression of genes associated with lipid metabolism and transport was detected using RT-qPCR to validate the transcriptional profiles from RNA sequencing. As expected, most of the genes tested by RT-qPCR had similar results as compared with RNA sequencing data. As shown in Fig. 3, feeding GN70 promoted the expression of FASN, but suppressed the expression of lipid oxidation (CPT1A, CPT1B, CPT2) and degradation (GK, MGLL, LIPG). Additionally, the expression of APOA2 was increased in GN70 group.

## Discussion

Previous studies reported that forage: concentrate ratios might affect hepatic lipid metabolism, resulting in altered levels of available energy for weight gain [29, 30]. In the modern intensive livestock systems in China, high concentrated rations (far over 60%) are widely used to provide greater energy. High concentrate diets and lack of exercise are important factors leading to excessive lipid steatosis and fat accumulation in the liver [14]. However, the molecular mechanism of how a high concentrate diet leads to hepatic lipid metabolic disorder is currently unavailable. The current study, using 60% dietary concentrate levels as the control, emphasized lipid metabolism regulatory network (lipid synthesis, decomposition, and transport) status to study the mechanisms of hepatic lipid metabolism affected by an increased concentrate diet in fattening lambs.

Bodyweight is one of the most important indexes that reflect animal growth and health condition. The similar ADG and F/G between two groups in this study were consistent with a previous study that Brand et al. [31] found no significant correlation between dietary energy level and performance (ADG and F/G) under long-term feeding. The unchanged performance with increasing concentrate may have resulted from the fact that the nutritional requirements of the lambs have been met in all the experimental diets. In our study, the daily digestible energy (DE) and crude protein (CP) received by GN60 and GN70 lambs far exceeded the nutritional requirements of 8 MJ DE and 95 g CP recommended in the Feeding Standard of Meat-producing Sheep and Goats of China (2004). Moreover, it has been reported that a slight increase in diet energy level had no significant effect on daily gain [32]. In our study, the diet provided 0.7 MJ/kg DE increase when increasing 10% concentrate from GN60 to GN70, which can be considered as a slight increase in energy.

It is worth noting that Brand et al. [33] found when the energy content of experimental diets exceeds animal requirements, the metabolism and growth of some tissue were affected. Expectedly, biochemical indices served as indicators and were used to monitor the physiological status, providing evidence regarding the metabolism change for animals in this study. The concentration of liver TG is a common biomarker for lipid metabolism. Under normal circumstances, liver stores only small amounts of triglyceride. The triglyceride accumulation within hepatocytes arises from an imbalance between lipid acquisition (i.e., fatty acid uptake and de novo lipogenesis) and removal (export as a component of VLDL particles), which is the hallmark of lipid deposition in liver [34]. In this study, TG which was an essential marker of lipid metabolism was increased in the liver of GN70 group. Within normal physiological ranges, transport rates of TG are consistent with the synthesis rates of TG as a protective response to hepatic metabolic capacity. The increased TG concentration in liver from GN70 lambs with an experimental period of 3 months suggested the synthetic rate of TG was higher than the degradation rate, indicating hepatic lipid metabolic abnormalities of fattening lambs. Interestingly, it has been shown that different animal species had variable metabolic responses in particular on lipid metabolism when the animals underwent excessively high concentrate diets. For instance, a study on dairy cows showed that feeding a high concentrate diet reduced liver TG levels but had a less potent effect on blood TG content [35]. However, Grum et al. [36] have reported that feed high concentrate diets did not affect liver TG of cows. Moreover, Dong et al. [12] reported that blood and hepatic TG levels were elevated in lactating goats fed high concentrate diets. Indeed, it has been reported that there were differences in liver lipid metabolism between species [37, 38]. The variable response to high concentrate diets in different animal species may have resulted from the reason that complex interactions among factors regulate the hepatic lipid metabolism, and the mechanisms underlying need to be further investigated.

Since hepatic lipid metabolism is tightly regulated by multiple interrelated genes, RNA sequencing was used to identify DEGs and to explore the critical pathways in the liver when fed a high concentrate diet using fattening lambs. Clear separation between GN60 and GN70 groups in liver transcriptome PCA showed alteration in hepatic transcription. The majority of enriched KEGG pathways were related to lipid metabolism, including PPAR signaling pathway, fatty acid metabolism, fatty acid degradation, and fatty acid biosynthesis. Enriched GO items of DEGs were positive regulation of triglyceride catabolic process, positive regulation of lipoprotein lipase activity, and positive regulation of lipid catabolic process. Likewise, the critical modules in PPI network were fatty acid degradation and cholesterol metabolism. The result of GO, KEGG pathway, and PPI analysis revealed the mechanism occurring with lipid transport and metabolism related pathway. The up-regulated FASN in GN70 implied more *de novo* lipogenesis in lambs. A similar result has also been reported by Dong et al. [12], who found an up-regulated expression of FASN in hepatic tissues of goats after being fed a high concentrate diet for 10 weeks. ACSL3 is a member belonging to the long-chain acyl-CoA synthetases (ACSLs) family, which is involved in cellular absorption of fatty acids and plays a crucial role in the synthesis of lipid droplets [39]. Up-regulated ACSL3 indicated increased TG synthesis in the liver of GN70 [40]. Moreover, the process of fatty acid degradation of the GN70 group has changed. Generally, ACSL1 accounts for 90% of the total ACS activity and is located on the outer mitochondrial membrane where it interacts with CPT1 and directs FAs towards mitochondrial, in which CPT2 participates in β-oxidation [41–43]. In the present study, down-regulated ACSL1, CPT1A, CPT1B and CPT2 in the hepatic tissue from GN70 group suggested that fatty acid transport and fatty acid oxidation were restrained. This was contrary to the observations of H Dong et al. [5], who observed a higher expression of ACSL1 in lactating goats fed a high concentrate diet, and the expression of CPT1 and CPT2 had no difference compared with goats fed a low concentrate diet. These differences could be explained by the fact that high concentrate diets increased lactating goat milk production, and the milk fat synthesis mobilized liver fat metabolism, thereby enhancing fatty acid oxidation [12]. GK is a critical gene for glycerol utilization, and its overexpression increases the consumption of sugars and triglycerides by cells [44]. MGLL plays an essential role in fatty acid metabolism, converting monoacylglycerides to free fatty acids and glycerol [45]. A similar function of phospholipase activity and triglyceride lipase activity has also been proposed for LIPG [46, 47]. Thus, dramatically descend expressional levels of GK, MGLL and LIPG indicated the attenuation of glycerolipid degradation in the hepatic tissue from GN70.

As expected, the expressions of some lipid transport related genes were changed in GN70. APOA2 and APOA5 are members of the apolipoprotein family closely linked with TG metabolism [48, 49]. In the current study, the expression of APOA2 was up-regulated while the expression of APOA5 was down-regulated. Nevertheless, it has been reported that the expression of APOA2 and APOA5 were considered to have a positive association with TG accumulation in the liver [49, 50]. Moreover, SA van den Berg et al. [51] reported that hepatic TG content was 50% higher in high-fat diet Apoa5^−/−^ mice compared with Wild-type (WT) mice. It was assumed that APOA5 may regulate TG metabolism through different pathways in different environments [51]. In blood, APOA5 was presumed as the only lipoprotein that lowers blood TG, while overexpression of APOA2 was positively associated with TG level [48, 52]. However, in the present study, the blood TG level was not different between GN60 and GN70. It is well known that TG is generally exported as constituents of VLDL into the blood circulation, the progress of which needs apoB package TG to create VLDL particles [53, 54]. No change in APOB gene in GN70 indicated that there were no differences in the corresponding TC and LDL in the blood, resulting in aligned TG secretion rate of GN70 as compared to GN60. Taken together, these results showed enhanced fatty acid synthesis and TG synthesis, inhibited fatty acid transport and oxidation, and unchanged TG secretion which was explained by an improved triglyceride content in the liver.

## Conclusion

**Fig. 4:**
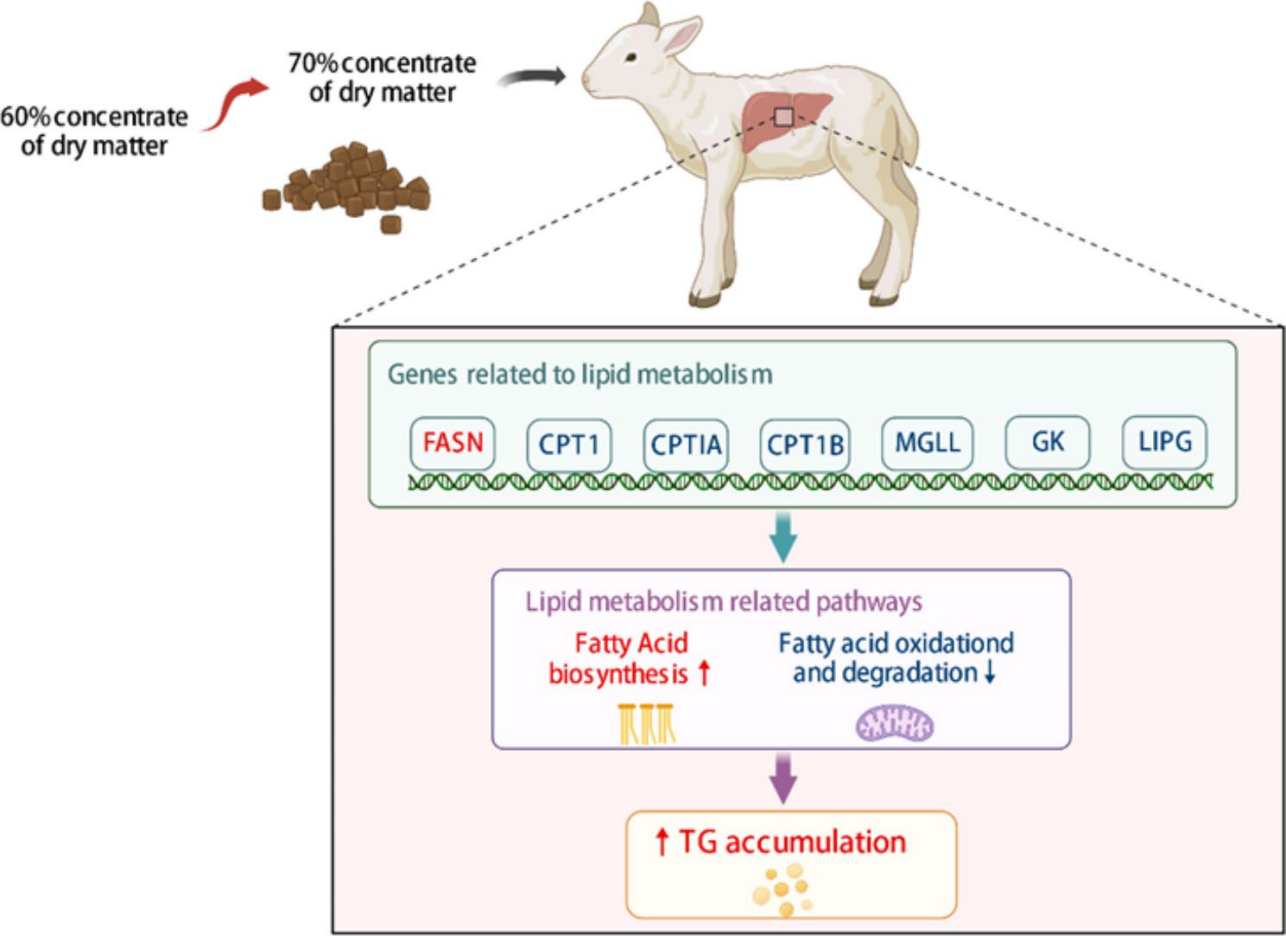
**Graphical abstract**: A systematic diagram illustrating the mechanism of increased dietary grain ratios influence on hepatic lipid metabolism disorders in fattened lambs. The red arrow indicates as activation and blue arrows indicates as suppression

In summary, our study revealed that a high concentrate diet induced lipid metabolism disorder in the livers of fattening lambs, leading to a significant accumulation of liver TG (Fig. 4). Transcriptome analysis showed significant gene expressional changes which may be accountable for the ectopic lipid deposition in the liver from lambs fed a high concentrate diet. Combined the GO and KEGG enrichment and PPI analysis of DEGs in hepatic transcriptome, the study revealed lipid transport and metabolism related pathway was differentially regulated in response to the high concentrate diet. Furthermore, the up-regulated FASN, ACSL3 indicated increased TG synthesis in the liver. Meanwhile, the down-regulated ACSL1, CPT1A, CPT1B and CPT2 suggested that fatty acid transport and fatty acid oxidation were restrained in GN70 group. Decreased expression levels of GK, MGLL and LIPG indicated the attenuation of glycerolipid degradation. The DEGs results suggest the increased hepatic TG synthesis and decreased hepatic fatty acid oxidation resulted in TG accumulation since the transport rates of TG were lower than the synthesis rates of TG. This study offers important knowledge for understanding the mechanisms of hepatic lipid metabolism in response to high concentrate diets for fattening lambs.

## Electronic supplementary material

Below is the link to the electronic supplementary material.

**Table S1**. Ingredient and chemical composition of diet fed during the experiment (DM basis)

**Table S2** Primer sequences used for real-time quantitative PCR

Hepatic transcriptome profiles of fattening lambs

The gene list of expressed in all animals (FPKM greater than 1)

Lists of dietary dependent genes fed diets

The gene list of differentially expressed genes (DEGs) between two groups

**Fig.S1** GO enrichment analysis of DEGs between GN60 and GN70. **Fig.S2** Protein?protein interaction (PPI) network analysis. (A) PPI network for DEGs. The Top3 highly connected clusters through MCL clustering, including (B) DNA replication, (C) Fatty acid degradation, (D) Cholesterol metabolism.

## Data Availability

The liver transcriptome data from current study have been submitted to the Sequence Read Archive (SRA) database (http://www.ncbi.nlm.nh.gov/sra) and the data are accessible through SRA Series accession number PRJNA794866 (https://www.ncbi.nlm.nih.gov/bioproject/PRJNA794866).

## Abbreviations

F: C:The ratio of concentrate to forage
TG: Triglycerides
TC: Cholesterol
RNA-seq: RNA-sequencing
IBW: initial body weight
ADFI: Average daily feed intake
ADG: Average daily gain
GLU: Glucose
LDL-C: Low-density lipoprotein cholesterol
HDL-C: High-density lipoprotein cholesterol
NEFA: Non-esterified fatty acid
BHBA: β-hydroxybutyric acid
DNB: DNA nanoball
FPKM: Transcription fragment per million fragments per thousand bases
DEGs: Differentially expressed genes
FDR: False discovery rate
F/G: Feed to gain ratio
DAVID: Database for Annotation, Visualization and Integrated Discovery
PCA: Principal component analysis
