# Supplementary material for: Genome wide transcriptome analysis provides bases on hepatic lipid metabolism disorder affected by increased dietary grain ratio in fattening lambs
Source: BMC Genomics. 2023 Jun 29;24:364. doi: 10.1186/s12864-023-09465-4 (PMC10308664; doi:10.1186/s12864-023-09465-4)
Supplement: Supplementary file 1 — Table S1. Ingredient and chemical composition of diet fed during the experiment (DM basis) [file 12864_2023_9465_MOESM1_ESM.docx]

**Table S1.** Ingredient and chemical composition of diet fed during the experiment (DM basis)

| Item | GN60^1^ | | GN70^2^ |
| --- | --- | --- | --- |
| Ingredients % | |  | |
| Alfalfa hay | | 40.00 | 30.00 |
| Corn | | 25.01 | 30.01 |
| Barely | | 5.87 | 10.87 |
| Soybean meal | | 12.5 | 12.5 |
| Wheat bran | | 8.72 | 8.72 |
| Cottonseed meal | | 3.00 | 3.00 |
| Beet molasses | | 1.00 | 1.00 |
| Sodium bicarbonate | | 0.70 | 0.70 |
| Calcium carbonate | | 0.50 | 0.50 |
| Sodium chloride | | 0.50 | 0.50 |
| Magnesium oxide | | 0.20 | 0.20 |
| ^3^Premix | | 2.00 | 2.00 |
| ^4^Chemical compositions, % | |  |  |
| Dry matter | | 89.21 | 88.96 |
| Digestible energy (MJ/kg) | | 12.17 | 12.70 |
| Crude protein | | 18.66 | 17.76 |
| Neutral detergent fiber | | 27.51 | 24.38 |
| Acid detergent fiber | | 18.04 | 15.05 |
| Crude fat | | 2.58 | 2.59 |
| Calcium | | 0.50 | 0.64 |
| Phosphorus | | 0.40 | 0.39 |

^1^ GN60 = 60% concentrate of dry matter.

^2^ HCD = 70% concentrate of dry matter.

^3^ GN60 contained per kg of diet: 35.4 g FeSO4•7H2O; 16.6 g ZnSO4•H2O; 3.3 g CuSO4•5H2O; 11.5 g MnSO4•H2O; 104.2 g MgSO4•H2O; 9.0 g Na2SeO3; 6.5 g KI; 2.8 g CoCl2•6H2O; 25.0 g Vitamin.

^4^ Chemical compositionswere estimated from the current diets.
