# Supplementary material for: Genome wide transcriptome analysis provides bases on hepatic lipid metabolism disorder affected by increased dietary grain ratio in fattening lambs
Source: BMC Genomics. 2023 Jun 29;24:364. doi: 10.1186/s12864-023-09465-4 (PMC10308664; doi:10.1186/s12864-023-09465-4)
Supplement: Supplementary file 2 — Table S2 Primer sequences used for real-time quantitative PCR [file 12864_2023_9465_MOESM2_ESM.docx]

Table S2 Primer sequences used for real-time quantitative PCR

| Gene^1^ | Primer sequences (5'-3')^2^ | Product size(bp) | Gene Bank |
| --- | --- | --- | --- |
| ACSL1 | F: GTTTCCAGAGGGGCATAC | 270 | XM_ 015104562.2 |
|  | R: CTTCAGTTCCGAGGGTGT |  |  |
| ACSL3 | F：AGAAGCCGAAGACCAC | 108 | XM_042244308.1 |
|  | R: TAGCATACAGCGTAACAAG |  |  |
| APOA2 | F: ATTGACTGTGCTGCTCC | 198 | XM_004002693.5 |
|  | R: TCCTCCTGCGTCTTCT |  |  |
| APOA5 | F: GATTGCCGCCTTCACTCA | 119 | XM_ 027979224.1 |
|  | R: TGCCTCTGTTGGCTTGG |  |  |
| CPT1A | F: CTTCCCATTCCGCACTTT | 167 | XM_015102894.3 |
|  | R: TCTCTGTTCTGCCCTCTCG |  |  |
| CPT1B | F: CAGCCGAACGAAGACC | 156 | NM_001009259.1 |
|  | R: AGCCAGGAAAGGGGAC |  |  |
| CPT2 | F: GGTGGCAACAAGGAGAC | 275 | XM_ 004001991.4 |
|  | R: TGGGGAGTGATAGCAGG |  |  |
| EBP | F: AGGGAGACAGCCGATAC | 154 | XM_027963097.2 |
|  | R: CCGACAGAGACCACAAG |  |  |
| FASN | F: CGGGAAGGGTGTTGAC | 132 | AF479289.1 |
|  | R: CCCAGAGGGTGGTTGT |  |  |
| GK | F: TGTCAGTAACCAGAGGGAAA | 178 | XM _ 004021983.4 |
|  | R: ATAAGTGCTAAGTGGAAGGC |  |  |
| LIPG | F: TGAATCAGGACAAGCCGAGC | 268 | NM_001308006.2 |
|  | R:GTGCCATAAAGGGTGACATAAAAG |  |  |
| MGLL | F: TATGGTCCTCATCTCCCC | 222 | XM_ 015102506.2 |
|  | R: GTCTTGTCCTGGCTCTTG |  |  |
| TKFC | F:TGCAGAAGTACGGAAAGGC | 264 | XM_042238193.1 |
|  | R: AGATGGCACGCAGAATGG |  |  |
| β-actin | F: TGAACCCCAAAGCCAACC | 107 | NM_001009784.1 |
|  | R: AGAGGCGTACAGGGACAGCA |  |  |
| GAPDH | F: ACGCTCCCATGTTTGTGATG | 146 | NM_001190390.1 |
|  | R: CATAAGTCCCTCCACGATGC |  |  |

^1^ACSL1: Acyl-Coa Synthetase Long Chain Family Member 1; ACSL3: Acyl-Coa Synthetase Long Chain Family Member 3; APOA2: Apolipoprotein A2; APOA5: Apolipoprotein A5; CPT1A: Carnitine Palmitoyltransferase 1A; CPT1B: Carnitine Palmitoyltransferase 1B; CPT2: Carnitine Palmitoyltransferase 2; EBP: Ebp Cholestenol Delta-Isomerase; FASN: Fatty Acid Synthase; GK: Glycerol Kinase; LIPG: Lipase G, Endothelial Type;MGLL: Monoglyceride Lipase; TKFC: Triokinase And Fmn Cyclase.

^2^ F = Forward primer; R = Reversed primer.
