# Supplementary material for: Genome wide transcriptome analysis provides bases on hepatic lipid metabolism disorder affected by increased dietary grain ratio in fattening lambs
Source: BMC Genomics. 2023 Jun 29;24:364. doi: 10.1186/s12864-023-09465-4 (PMC10308664; doi:10.1186/s12864-023-09465-4)
Supplement: Supplementary file 7 — Fig.S1 GO enrichment analysis of DEGs between GN60 and GN70. Fig.S2 Protein?protein interaction (PPI) network analysis. (A) PPI network for DEGs. The Top3 highly connected clusters through MCL clustering, including (B) DNA replication, (C) Fatty acid degradation, (D) Cholesterol metabolism. [file 12864_2023_9465_MOESM7_ESM.docx]

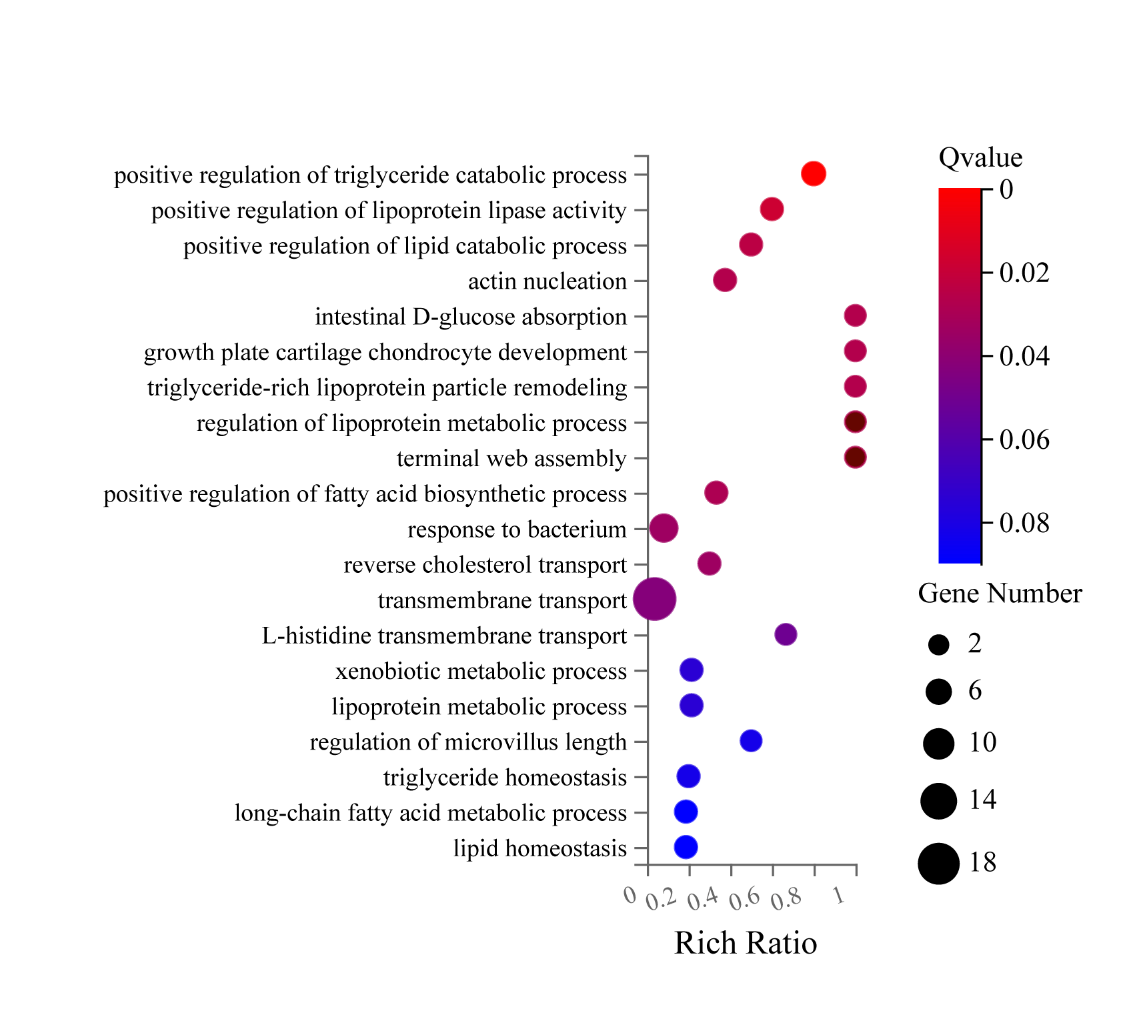


Fig.S1 GO enrichment analysis of DEGs between GN60 and GN70


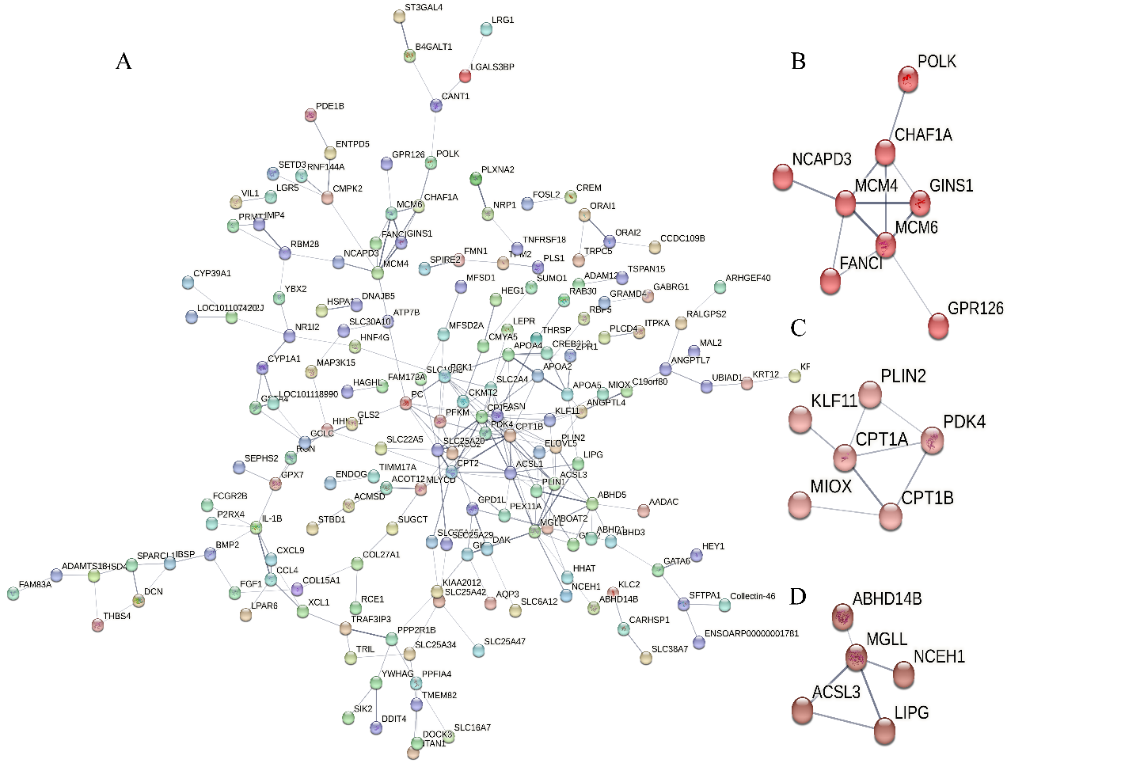


Fig.S2 Protein–protein interaction (PPI) network analysis. (A) PPI network for DEGs. The Top3 highly connected clusters through MCL clustering, including (B) DNA replication, (C) Fatty acid degradation, (D) Cholesterol metabolism.
